# Non-Selective Cation Channels Mediate Chloroquine-Induced Relaxation in Precontracted Mouse Airway Smooth Muscle

**DOI:** 10.1371/journal.pone.0101578

**Published:** 2014-07-03

**Authors:** Ting Zhang, Xiao-Jing Luo, Wen-Bo Sai, Meng-Fei Yu, Wen-Er Li, Yun-Fei Ma, Weiwei Chen, Kui Zhai, Gangjian Qin, Donglin Guo, Yun-Min Zheng, Yong-Xiao Wang, Jin-Hua Shen, Guangju Ji, Qing-Hua Liu

**Affiliations:** 1 Institute for Medical Biology & Hubei Provincial Key Laboratory for Protection and Application of Special Plants in Wuling Area of China, College of Life Sciences, South-Central University for Nationalities, Wuhan, China; 2 National Laboratory of Biomacromolecules, Institute of Biophysics, Chinese Academy of Sciences, Beijing, China; 3 Department of Medicine-Cardiology, Feinberg Cardiovascular Research Institute, Northwestern University Feinberg School of Medicine, Chicago, Illinois, United States of America; 4 Lankenau Institute for Medical Research & Main Line Health Heart Center, Wynnewood, Pennsylvania, United States of America; 5 Center for Cardiovascular Sciences, Albany Medical College, Albany, New York, United States of America; Temple University School of Medicine, United States of America

## Abstract

Bitter tastants can induce relaxation in precontracted airway smooth muscle by activating big-conductance potassium channels (BKs) or by inactivating voltage-dependent L-type Ca^2+^ channels (VDLCCs). In this study, a new pathway for bitter tastant-induced relaxation was defined and investigated. We found nifedipine-insensitive and bitter tastant chloroquine-sensitive relaxation in epithelium-denuded mouse tracheal rings (TRs) precontracted with acetylcholine (ACH). In the presence of nifedipine (10 µM), ACH induced cytosolic Ca^2+^ elevation and cell shortening in single airway smooth muscle cells (ASMCs), and these changes were inhibited by chloroquine. In TRs, ACH triggered a transient contraction under Ca^2+^-free conditions, and, following a restoration of Ca^2+^, a strong contraction occurred, which was inhibited by chloroquine. Moreover, the ACH-activated whole-cell and single channel currents of non-selective cation channels (NSCCs) were blocked by chloroquine. Pyrazole 3 (Pyr3), an inhibitor of transient receptor potential C3 (TRPC3) channels, partially inhibited ACH-induced contraction, intracellular Ca^2+^ elevation, and NSCC currents. These results demonstrate that NSCCs play a role in bitter tastant-induced relaxation in precontracted airway smooth muscle.

## Introduction

In 1867, Schofield RH discovered “taste-goblets” in cat and dog tongues [1], which were then named taste buds [2]. Taste buds contain different types of receptor cells that sense various tastes, such as bitter, sweet, sour, salty, and umami [3], [4]. Taste receptors type 2 (TAS2R) are responsible for detecting bitter sensation [5].

TAS2Rs have recently been found to be expressed in airway smooth muscle cells and bitter taste stimuli can affect airway muscle force [6]–[15]. These receptors mediate bitter tastant-induced relaxation in airway smooth muscle precontracted by muscarinic (M) receptor agonists. TAS2Rs can be activated by bitter tastants, once activated, they induce an increase in intracellular Ca^2+^ through the G_βγ_ protein-PLCβ-IP_3_-IP_3_R pathway. This Ca^2+^ increase then activates BKs, resulting in membrane hyperpolarization and partial relaxation [8], [10]. However, the bitter tastant chloroquine can inhibit BKs [9]. A recent study demonstrated that chloroquine-induced relaxation in precontracted airway smooth muscle is due to the inhibition of voltage-dependent L-type Ca^2+^ channels (VDLCCs) mediated by G proteins [16]. Therefore, the mechanism of bitter tastant-induced relaxation in precontracted airway smooth muscle remains unclear.

NSCCs represent a family of ion channels that generally conduct mono- (i.e., Na^+^, and K^+^) and divalent (i.e., Ca^2+^) cations with relatively poor discrimination. Thus, the activation of NSCCs results in Ca^2+^ influx-inducing contraction in muscle. In this study, we found that, in addition to VDLCCs, these NSCCs also play a role in bitter tastant-induced relaxation in precontracted airway smooth muscle.

## Materials and Methods

### Reagents

Fluo-4 AM and fura-2 AM were purchased from Invitrogen (Eugene, OR, USA). The other reagents were purchased from Sigma (St. Louis, MO, USA) and Tocris Bioscience (Bristol, UK). Niflumic acid, fluo-4 AM, and fura-2 AM were dissolved in DMSO, and other agonists and antagonists were dissolved in physiological saline solution (PSS). In single cell experiments, the reagents were locally delivered onto the cells through a 200 *μ*m diameter tube.

### Animals

Mature BALB/c male mice were purchased from Hubei Provincial Center for Disease Control and Prevention, Wuhan, China. Mice were housed under controlled temperatures (21–23°C) and light conditions (lights on 8∶00–20∶00) with *ad libitum* access to water and food.

This study was performed in strict accordance with the recommendations in the Guide for the Care and Use of Laboratory Animals of the National Institutes of Health. All experiments were approved by the Institutional Animal Care and Use Committee at the South-Central University for Nationalities (Permit number: 2012-QHL-1). Mice were sacrificed by intraperitoneal injection of sodium pentobarbital (150 mg/kg) and tissues were then taken.

### Force measurement in tracheal rings (TRs)

Muscle force was measured as previously described [17]. Briefly, mice were sacrificed following intraperitoneal injection of sodium pentobarbital (150 mg/kg), and tracheae were obtained and transferred to PSS (mM): 135 NaCl, 5 KCl, 1 MgCl_2_, 2 CaCl_2_, 10 HEPES, and 10 glucose (pH = 7.4). The epithelium-denuded TRs were prepared and mounted in a 10-mL organ bath chamber with a preload of 0.5 g. After a 60-min equilibration, the TRs were precontracted with ACH (10^−4^ M), washed, and rested for 3 times. Following an additional 30 min rest, the experiments were started.

### Isolation of single ASMCs

Single mouse ASMCs were enzymatically isolated as previously described [18]. Briefly, after the mice were sacrificed through an intraperitoneal injection of sodium pentobarbital (150 mg/kg), tracheae were removed and transferred to an ice-cold low-Ca^2+^ physiological saline solution (LCPSS) containing (mM) 135 NaCl, 5 KCl, 1 MgSO_4_, 10 glucose, 10 HEPES, and 0.1 CaCl_2_ (pH = 7.4). The epithelium-denuded trachealis tissues were minced and incubated for 20 min at 37°C in LCPSS containing 1 mg/mL papain, 0.5 mg/mL dithioerythritol, and 1 mg/mL bovine serum albumin (BSA). The partially digested tissues were transferred to LCPSS containing 1 mg/mL collagenase H, 1 mg/mL dithiothreitol, and 1 mg/mL BSA at 37°C for 20 min. The tissues were then washed 3 times and triturated in LCPSS to yield single ASMCs.

### Measurement of whole-cell intracellular Ca^2+^

Intracellular Ca^2+^ was measured and analyzed as previously described [18], with some modifications. We used an LSM 700 laser scanning confocal microscope (Carl Zeiss, Jena, Germany) and XY scanning to measure the fluorescence intensity of fluo-4 AM. A pinhole set at 1 AU (29.5 µm), scanning speed of 9, and a 40X/1.30 oil objective lens were used. Freshly isolated ASMCs were incubated with 2 µM fluo-4 AM for 15 min at room temperature in the microscope recording chamber and then superfused with 5% CO_2_/95% O_2_-bubbled PSS for 10 min. The excitation was provided at 488 nm and the emitted fluorescence of fluo-4 AM was imaged through a 505 nm filter. XY images were acquired and fluorescence intensity was analyzed using Zen 2010 software (Carl Zeiss, Jena, Germany).

Intracellular Ca^2+^ was measured using fura-2 AM as previously described [19], [20]. Cells were loaded with fura-2 AM (2.5 µM). Paired 340/380 fluorescence images were acquired using the TILL imaging system (FEI Munich GmbH, Germany), and ratios were calculated that were used to represent relative changes in intracellular Ca^2+^ concentration.

### Simultaneous measurements of the changes in intracellular Ca^2+^ and cell length

To measure changes in cell length [18], we used a 488 nm laser light as transmitted light and the transmitted images were generated by a photomultiplier tube (PMT). The transmit image and the fluo-4 AM fluorescence images of single ASMCs were simultaneously measured using an LSM 700 laser scanning confocal microscope and analyzed using Zen 2010 software (Carl Zeiss, Jena, Germany).

### Patch clamp

The ion channel currents were measured using an EPC-10 patch-clamp amplifier (HEKA, Lambrecht, Germany) [21], [22]. The ACH-induced NSCC currents were recorded with a ramp using a perforated whole-cell configuration with a holding potential of −60 mV. The ramp was performed over 500 ms from −80 to +60 mV. The values at −70 mV were used to represent the NSCC currents. The pipette solution consisted of (mM) 18 CsCl, 108 Cesium acetate, 1.2 MgCl_2_, 10 HEPES, 3 EGTA, and 1 CaCl_2_ (pH adjusted to 7.2 with Tris) [23]. The free Ca^2+^ concentration was approximately 70 nM, calculated using WEBMAXC (www.stanford.edu/~cpatton/webmaxc/webmaxcS.htm). The pipette solution contained 300 µg/mL nystatin for perforation of the membrane of smooth muscle cells. The bath solution was PSS as described above, but with K^+^ omitted. We added 10 mM tetraethylammonium (TEA) chloride, 100 µM niflumic acid, and 10 µM nifedipine to the bath solution to block the K^+^, Cl^−^, and VDLCC currents, respectively, to further purify NSCC currents.

Single NSCC currents were recorded using an outside-out approach. The single channel currents at −90 mV were acquired at a digitization rate of 4 kHz and filtered at 1 kHz. Events were detected and all-point amplitude histograms were plotted using the Clampfit 9 software (Axon Instruments, CA, USA). The histograms were fitted using the Gaussian distribution function, and amplitudes of single channels were obtained and used to calculate the single channel conductance (i.e., pS value). The pipette and bath solutions were similar as that previously used for single NSCC recordings with outside-out technique [24].

### Data analysis

The results are expressed as mean ± SEM. Comparisons between two groups were performed with Student’s *t*-test using Origin 9.0 software (OriginLab, Northampton, USA). Differences with *p*<0.05 were considered significant.

## Results

### Chloroquine blocks nifedipine-insensitive precontraction in mouse airway smooth muscle

**Figure 1:**
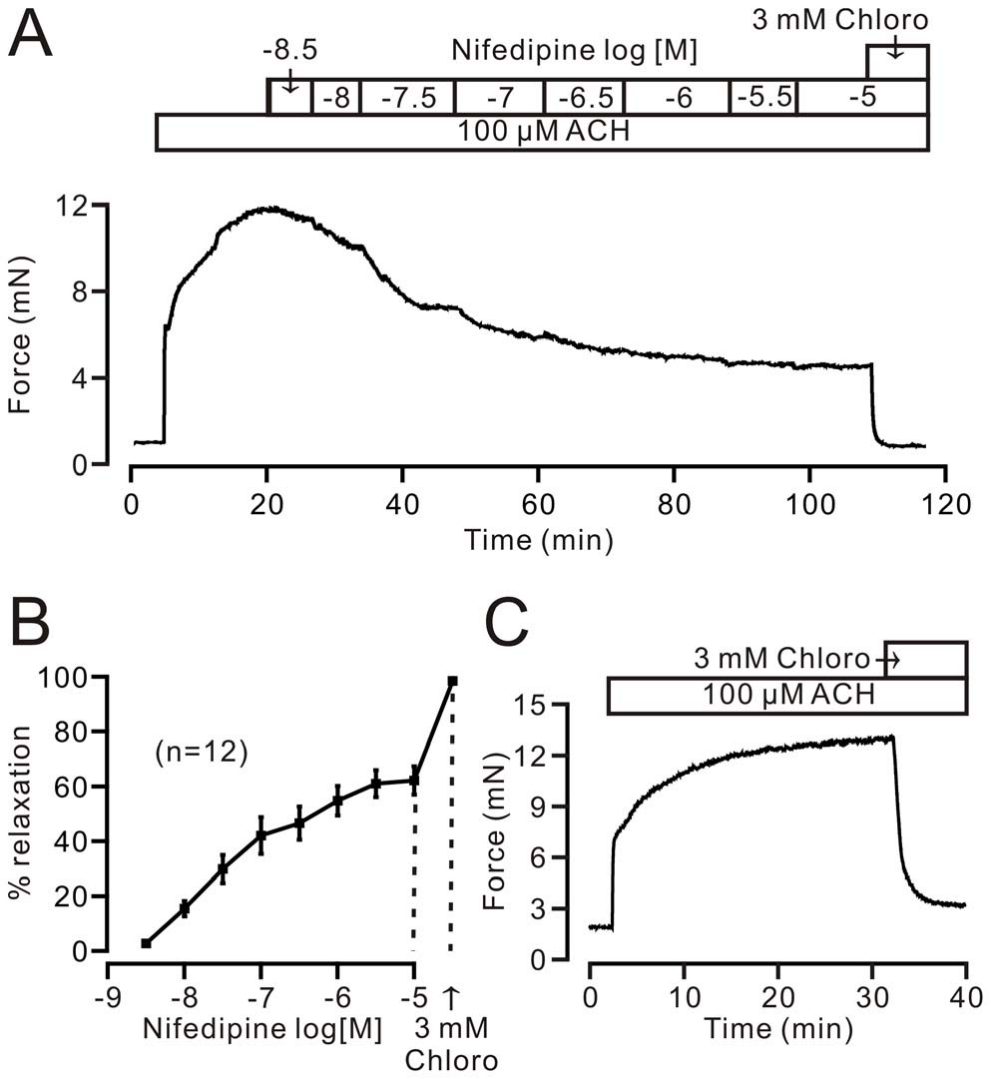
Nifedipine-insensitive pathway contributes to chloroquine-induced relaxation in ACH-precontracted mouse TRs. (A) Mouse TRs were precontracted by ACH. After contraction reached a steady-state, nifedipine was cumulatively added and induced relaxation. When the relaxation reached its maximum, chloroquine was added and the nifedipine-resistant contraction was inhibited. (B) Summary from 12 experiments shown in (A). (C) One representative of the 12 experiments for chloroquine-induced relaxation in ACH-precontracted mouse TRs. These results demonstrate that, in addition to VDLCCs, an unknown nifedipine-insensitive pathway plays a role in chloroquine-induced relaxation.

We first observed relaxation following blockade of VDLCCs in this study. TRs were contracted with ACH (100 µM). When contraction reached a steady-state, nifedipine, a selective blocker of VDLCCs, was cumulatively added to the organ chambers, which resulted in a series of relaxations (Figure 1A, B). When nifedipine (10 µM) induced maximal relaxation (66.2±5.2%, 12 rings/12 mice), chloroquine (3 mM) was added, which resulted in a maximal relaxation to 98.5±1.0% (Figure 1A, B). When only chloroquine (3 mM) was added, significant relaxation (85.5±2.6%, 12 rings/12 mice) was also observed (Figure 1C). These data suggest that chloroquine induces relaxation in precontracted mouse airway smooth muscle through both VDLCC-dependent and -independent pathways.

**Figure 2:**
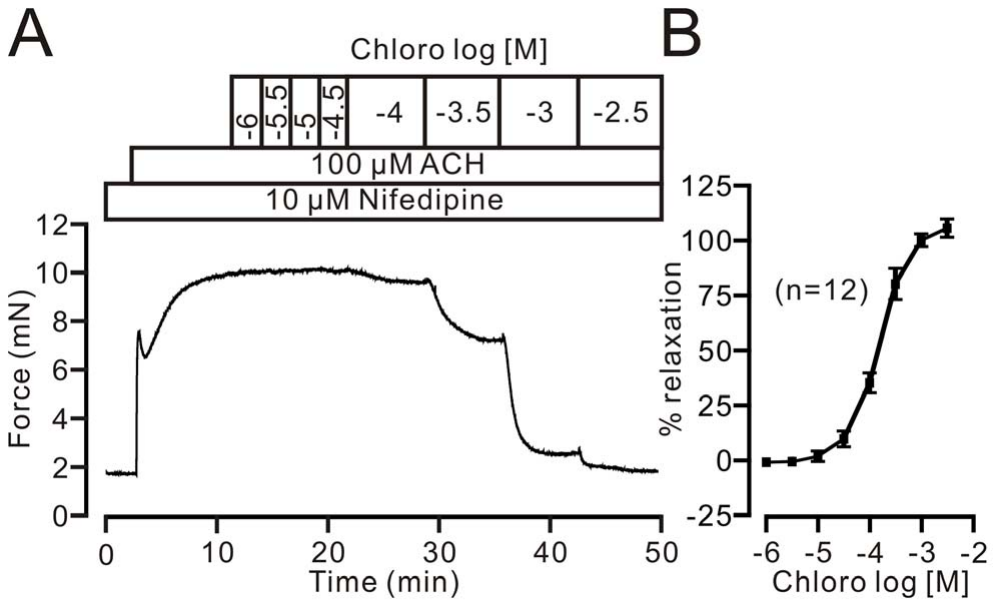
Chloroquine inhibits nifedipine-insensitive relaxation. (A) Chloroquine completely blocked ACH-induced precontraction. (B) Dose-response of chloroquine. These results also indicate that an unknown pathway contributes to chloroquine-induced relaxation.

To further confirm the existence of nifedipine-insensitive component, we conducted the following experiments. We used 10 µM nifedipine (a concentration that completely inhibits VDLCCs; cf Fig. 1A) to incubate the TRs for 10 min, which can completely inhibited VDLCC-mediated contraction, as shown in Figure 1A; we then observed the relaxant actions of chloroquine in ACH-induced precontraction (Figure 2). The ACH-induced steady-state contraction was gradually inhibited following cumulative addition of chloroquine; maximal relaxation was 105.0±4.2% (8 rings/8 mice) at 3.16 mM (i.e., Log_10_^−2.5^) chloroquine. These results further indicate that a VDLCC-independent pathway plays a pivotal role in chloroquine-induced relaxation.

### Chloroquine simultaneously inhibits Ca^2+^ elevation and cell shortening induced by ACH in single ASMCs

**Figure 3:**
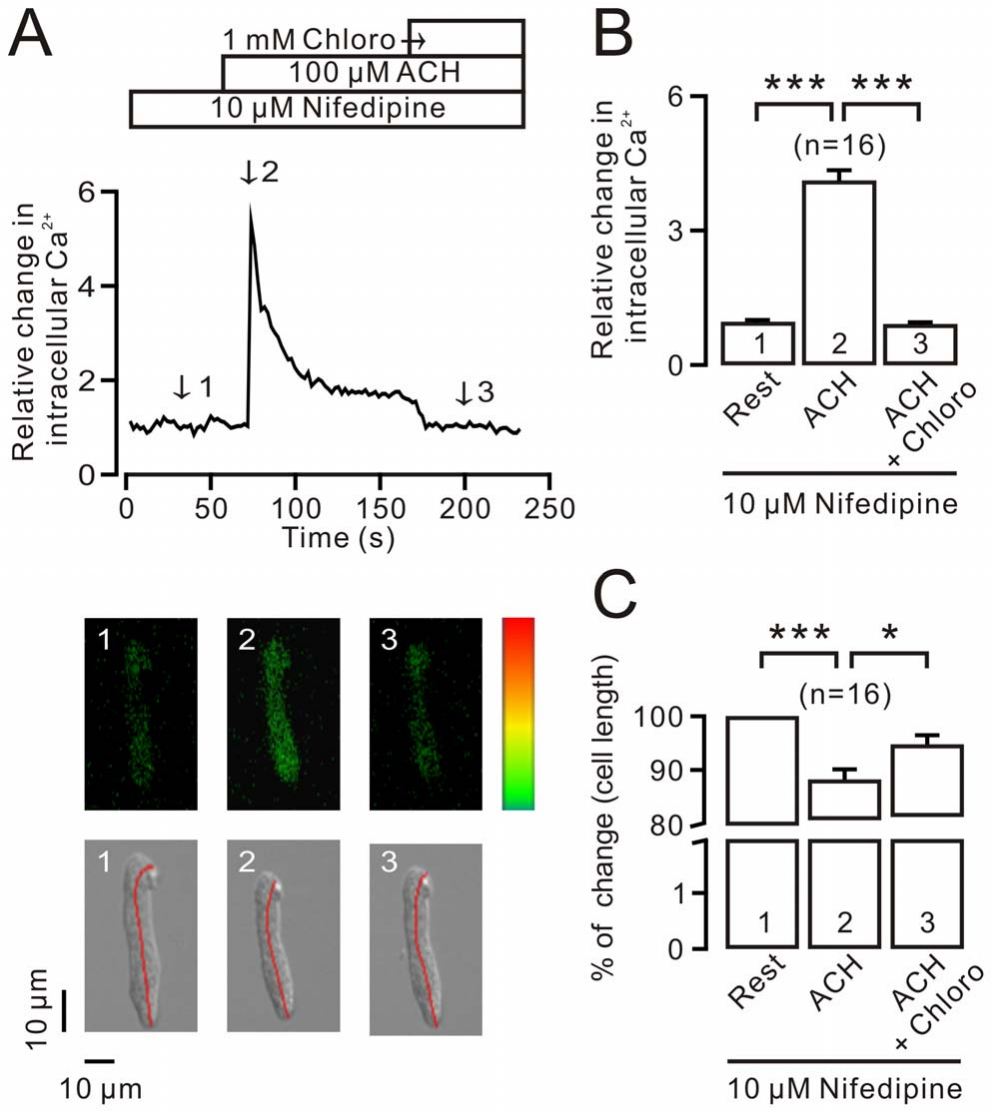
Chloroquine blocks the ACH-induced increase in Ca^2+^ and cell shortening in single ASMCs. (A) Single ASMCs were loaded with 2 µM fluo-4 AM. The intracellular Ca^2+^ and cell length were measured simultaneously in one cell. ACH evoked an increase in intracellular Ca^2+^, which was inhibited by chloroquine. Three fluorescence and transmitted images, indicated at the different time points numbered 1, 2, and 3, respectively, further reveal the Ca^2+^ changes and cell length alterations, respectively. (B, C) Summary of the average changes in the Ca^2+^ levels and cell lengths. *: *p*<0.05; ***: *p*<0.001.

To investigate the mechanism of the VDLCC-independent pathway in chloroquine-induced relaxation, we simultaneously observed chloroquine-induced changes in intracellular Ca^2+^ and cell length in fluo-4 AM loaded ASMCs (Figure 3A). Following the application of 100 µM ACH, intracellular Ca^2+^ sharply increased, quickly decreased, and then maintained a plateau. The Ca^2+^ plateau was completely inhibited following the addition of 1 mM chloroquine (*upper* in Figure 3A, a concentration was used in the following experiments performed on single cells). The Ca^2+^ level at resting state, peak, and sustained phrase were indicated by numbers 1, 2, and 3 and the corresponding cell lengths were indicated using three single images (*bottom* in Figure 3A). The mean Ca^2+^ levels (Figure 3B) and change in cell lengths (Figure 3C) from 16 cells at these three time points were calculated. These results indicate that the VDLCC-independent pathway-mediated relaxation in precontracted airway smooth muscle is due to a decrease in intracellular Ca^2+^.

### Chloroquine induces an inhibition of Ca^2+^ influx

**Figure 4:**
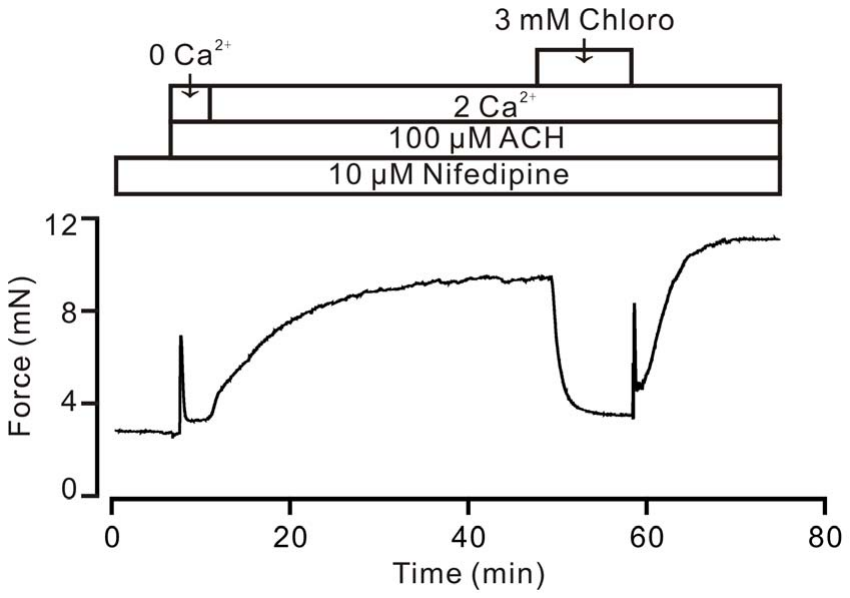
Chloroquine inhibits Ca^2+^ influx. Under Ca^2+^-free conditions (0 Ca^2+^ and 0.5 mM EGTA), ACH induced a fast transient contraction. Following the addition of 2 mM Ca^2+^, a larger contraction occurred and was reversibly inhibited by chloroquine. These results demonstrate that chloroquine induces relaxation by blocking ACH-induced Ca^2+^ entry.

To further investigate a reduction in intracellular Ca^2+^ induced by chloroquine, the following experiments were conducted. Under Ca^2+^-free conditions (0 Ca^2+^ and 0.5 mM EGTA), ACH was added, which triggered a transient contraction through intracellular Ca^2+^ release. When the contraction reached a steady-state, 2 mM Ca^2+^ was restored in the organ chamber and a strong and sustained contraction subsequently occurred. Following the addition of chloroquine (3 mM), the contraction was inhibited by 104.9±3.0% (8 rings/8 mice), and the contraction was reversed following washout (Figure 4). These experiments demonstrate that the chloroquine-induced intracellular Ca^2+^ decrease is due to inhibition of Ca^2+^ influx, which will be responsible for VDLCC-independent pathway-mediated decrease in intracellular Ca^2+^ and relaxation in precontracted airway smooth muscle.

### Chloroquine inhibits whole-cell NSCC currents

**Figure 5:**
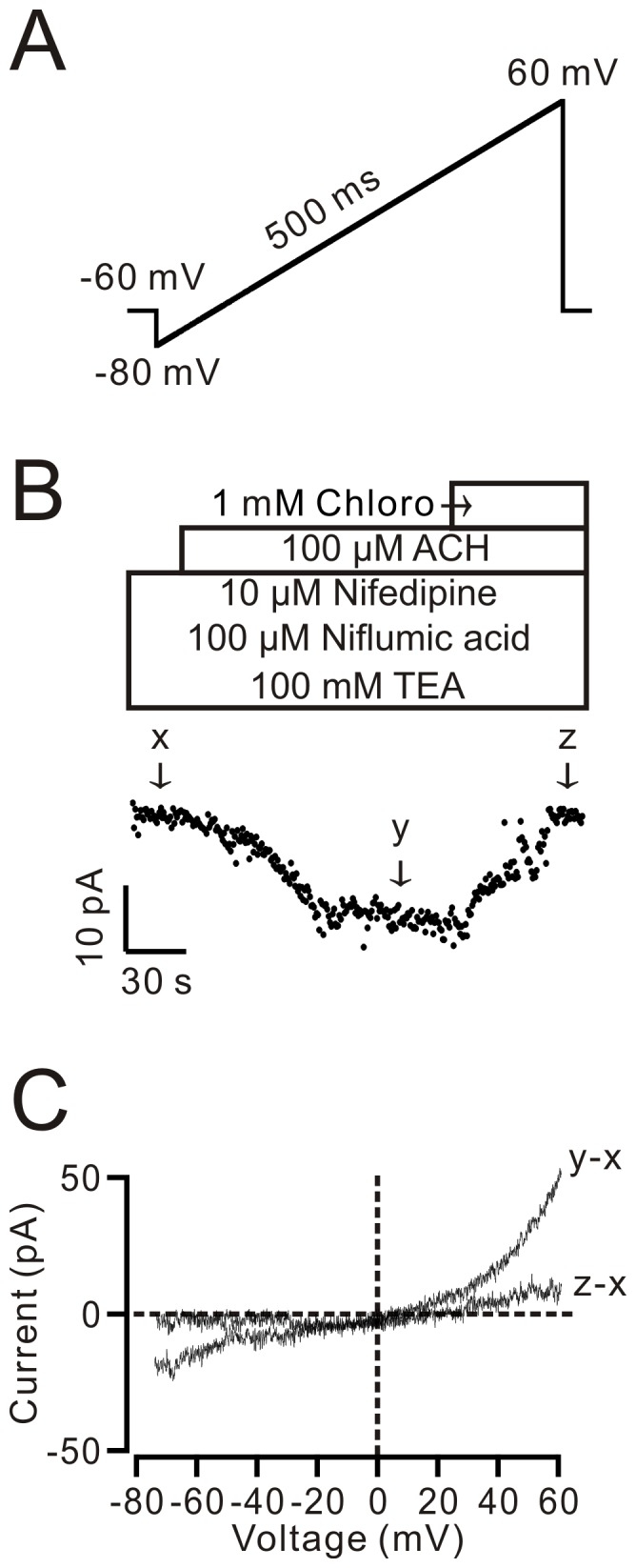
Chloroquine induces inhibition of ACH-induced NSCC currents. (A) The ramp used for recording the NSCC currents. (B) ACH induced NSCC currents at −70 mV that was blocked by 1 mM chloroquine. (C) Representatives of net ramp currents at time points y and z (the leak currents at time point x were subtracted). These results indicate that chloroquine can inhibit NSCCs.

To define the chloroquine-inhibited Ca^2+^ influx pathway, we studied the effect of chloroquine on ACH-induced NSCC currents, as ACH activates NSCCs through muscarinic (M) receptors, resulting in intracellular Ca^2+^ increase [25], [26]. We used a ramp (Figure 5A) to record the NSCC currents. The current values at −70 mV were chosen to represent the ACH-activated NSCC currents. NSCC currents (peak currents were 18.0±3.3 pA, n = 7) were induced by ACH and completely inhibited following the application of 1 mM chloroquine (Figure 5B). Representative ramp currents are depicted in Figure 5C. The reversal potential for ACH-induced currents was −0.11±0.35 mV (n = 7), which is close to 0 mV, further indicating these currents are NSCC currents. These data suggest that chloroquine results in the inhibition of NSCCs.

### Chloroquine inhibits single NSCC activity

**Figure 6:**
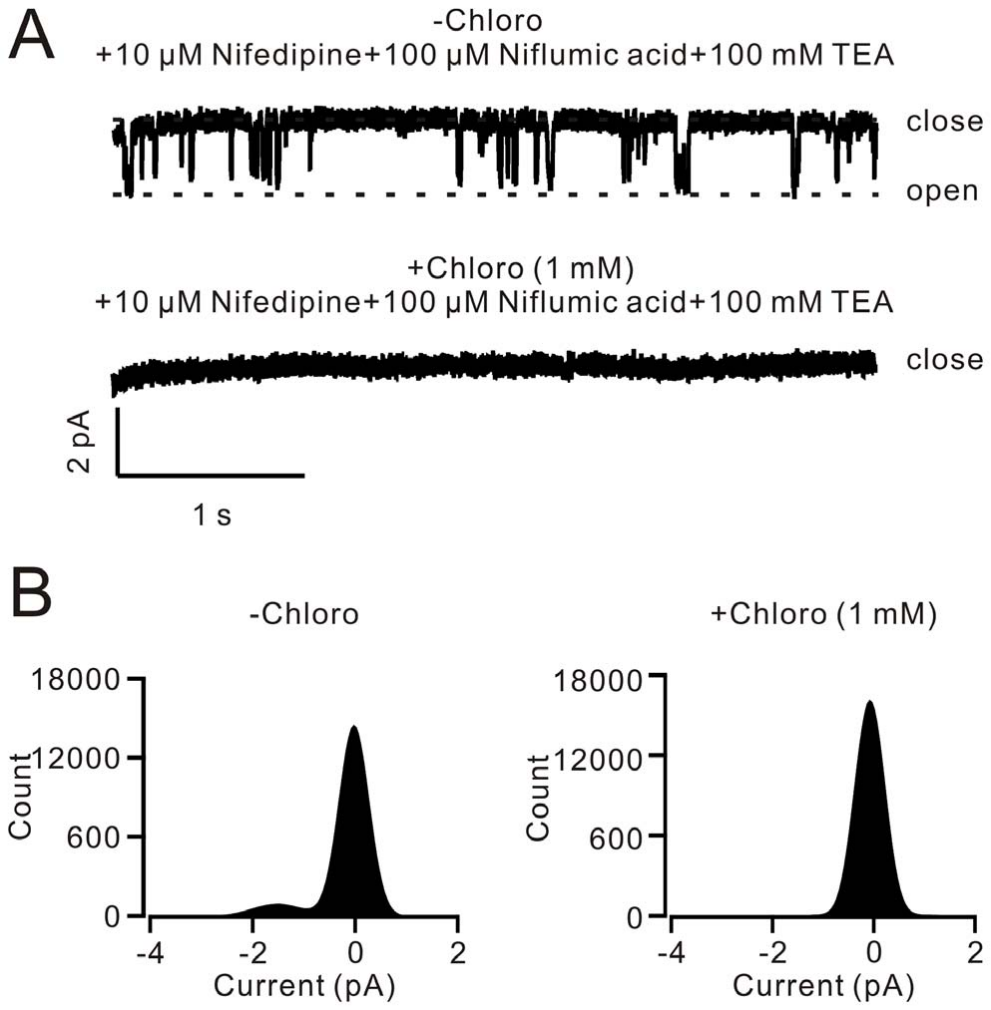
Chloroquine blocks single NSCC currents in excised patches. (A) Single NSCC currents at −90 mV were measured using the outside-out approach. The currents were blocked by 1 mM chloroquine. (B) The amplitude distributions of the currents, which were used to calculate single NSCC conductance that was 18.1±0.8 pS (n = 6). These results imply that chloroquine directly blocks NSCCs.

In order to further explore the mechanism of chloroquine-induced inhibition of NSCCs, we observed the effect of chloroquine on single NSCC activity. Single NSCC currents were recorded at −90 mV using the outside-out configuration, which were completely blocked by 1 mM chloroquine (Figure 6A). NSCC conductance was calculated on the basis of the amplitude distribution (Figure 6B), which was 18.1±0.8 pS (n = 6). This value is close to 14.8 pS measured in guinea-pig ventricular cells [27] and 23.0 pS in rabbit portal vein myocytes [24]. These results indicate that chloroquine can directly block NSCCs.

### Pyr3 inhibits ACH-induced contraction, Ca^2+^ rise, and NSCC currents

**Figure 7:**
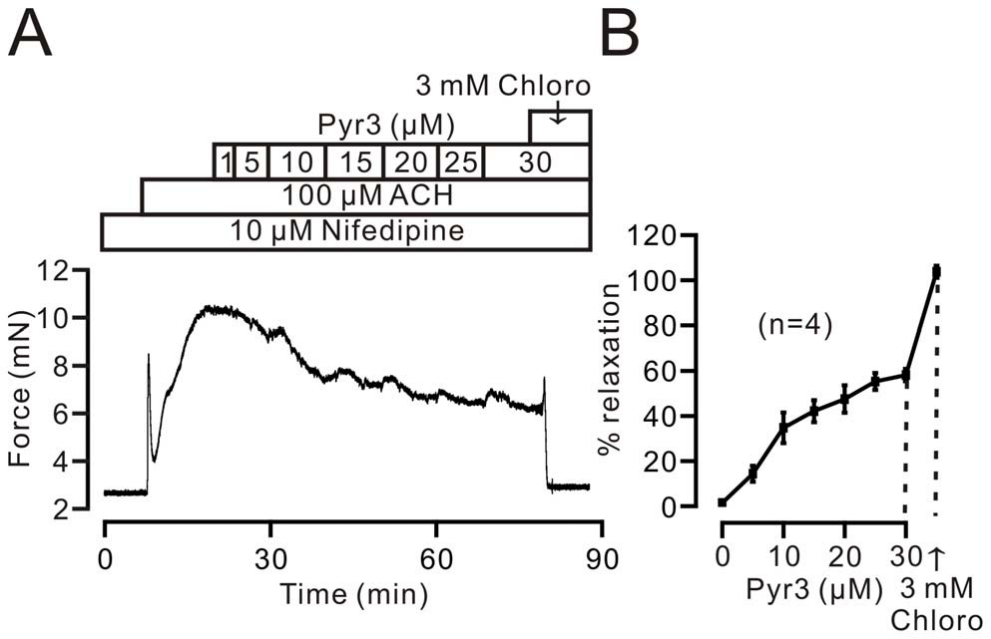
Pyr3 inhibits ACH-induced contraction. (A) ACH induced a contraction in a dose-dependent manner, which was inhibited by Pyr3, a blocker of TRPC3 channels. The remained contraction was completely blocked by 3 mM chloroquine. The dose-response curve is shown in (B). This result demonstrates that TRPC3 channels play a partial role in chloroquine-induced relaxation.

**Figure 8:**
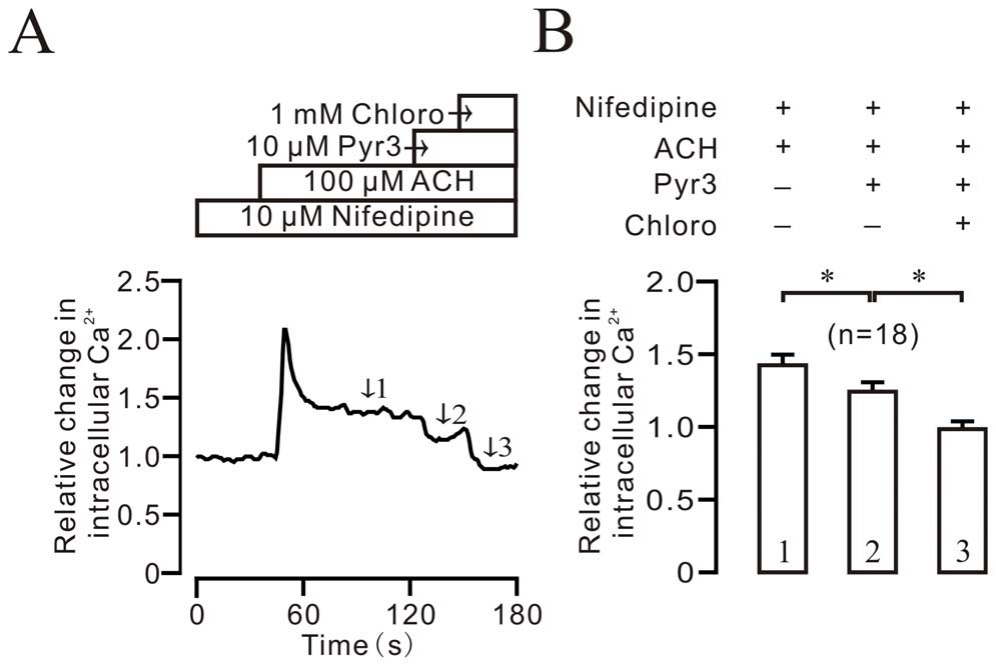
Pyr3 inhibits ACH-induced intracellular Ca^2+^ elevation. (A) ACH-induced sustained Ca^2+^ rise was partially inhibited by 10 µM Pyr3, and the Pyr3-resistant component was blocked by 1 mM chloroquine. (B) Summary of Pyr3- and chloroquine-induced Ca^2+^ decrease in 18 cells, respectively. *: *p*<0.05.

To further define the role of NSCCs, we used pyrazole 3 (Pyr3, 10 µM) to inhibit TRPC3 channels [28], which are one type of NSCCs, and observed its effect on the responses induced by ACH. ACH (100 µM) induced a typical steady-state contraction in TRs, which was inhibited by Pyr3 in a dose-dependent manner. When the Pyr3-induced inhibition reached a maximum, chloroquine (3 mM) was added and reduced contraction to baseline (Figure 7A). The dose-response curve was plotted based on the similar experiments in 4 TRs/4 mice (Figure 7B). Pyr3, at 30 µM, induced maximal inhibition (56.8±2.8%). These results were also supported by Ca^2+^ measurements using fura-2 AM dye. As shown in Figure 8A, ACH induced a Ca^2+^ increase, which was partially inhibited by Pyr3 (10 µM), and the Pyr3-resistant component was blocked by chloroquine (1 mM). The Ca^2+^ levels at the time points indicated by number 1, 2, and 3 were analyzed as shown in Figure 8B. These data indicate that the inactivation of TRPC3 channels plays a role in chloroquine-induced relaxation in precontracted airway smooth muscle.

**Figure 9:**
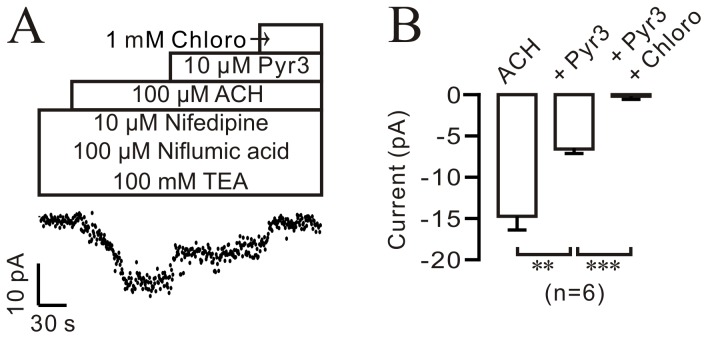
Pyr3 blocks ACH-activated NSCC currents. (A) ACH-activated NSCC currents were partially inhibited by 10 µM Pyr3 and the remaining currents were totally blocked by chloroquine. (B) Summary of the inhibitory effect of Pyr3 and chloroquine on ACH-induced NSCC currents (n = 6 cells/4 mice). This result suggests that NSCC currents inhibited by chloroquine were partially from TRPC3 channels. **: *p*<0.01; ***: *p*<0.001.

To further support these findings, we recorded ACH-activated NSCC currents. We found that the currents were partly blocked by Pyr3 (10 µM) and the remaining components were inhibited by chloroquine (1 mM). Pyr3 and chloroquine decreased the currents from −14.74±1.66 pA to −6.59±0.65 pA and −0.19±0.07 pA (n = 6 cells from 4 mice), respectively (Figure 9). These results further suggest that inactivation of TRPC3 channels play a partial role in chloroquine-induced relaxation.

## Discussion

In the present study, our data demonstrated that chloroquine can induce relaxation in precontracted airway smooth muscle through inhibition of VDLCCs and NSCCs. The mechanism of inhibition on NSCCs is due to direct blockade by chloroquine.

M receptor agonists can induce the activation of both VDLCCs and NSCCs, which in turn lead to Ca^2+^ influx to increase intracellular Ca^2+^, triggering a contraction in airway smooth muscle [16], [25], [26]. In this study, we used the M receptor agonist ACH to precontract TRs. We found chloroquine can inhibit nifedipine-insensitive relaxation (Figure 1A,B). It has previously been reported that chloroquine can result in inactivation of VDLCCs [16], hence, in this study, we only focused on defining the pathway that was insensitive to nifedipine and sensitive to chloroquine (Figures 1A,B and 2). Thus, all experiments were done in the presence of 10 µM nifedipine.

One cause of relaxation is intracellular Ca^2+^ decrease. Thus, we found that chloroquine inhibited the ACH-induced Ca^2+^ rise and corresponding cell shortening (Figure 3). The Ca^2+^ decrease was due to chloroquine blocking Ca^2+^ influx, as chloroquine can reversibly block Ca^2+^ influx-induced contraction (Figure 4).

We then defined which pathway was responsible for the Ca^2+^ influx. Previous studies have demonstrated that the activation of M receptor results in the activation of NSCCs in ASMCs. The activated NSCCs in turn mediate Ca^2+^ influx, leading to a rise in cytosolic Ca^2+^ [25], [26]. Therefore, we measured the ACH-induced NSCC currents and found that these currents were inhibited by 1 mM chloroquine (Figure 5). This suggests that inactivation of NSCCs might be the reason for the chloroquine-induced relaxation. Moreover, our results (shown in Figure 6) indicated that chloroquine can directly block NSCCs because it can block single NSCC currents.

Since TRPC1-7 are NSCCs, we used Pyr3, a blocker of TRPC3, to test whether these channels are involved in chloroquine-induced relaxation [29]. We found that Pyr3 caused a partial relaxation in ACH-precontracted mouse airway smooth muscle (Figure 7), suggesting that TRPC3 is one type of NSCC. These results were further confirmed by Ca^2+^ measurement (Figure 8) and whole-cell NSCC current recordings (Figure 9); however, we can not exclude STIM1/Orai1 channels because these channels are also inhibited by Pyr3 [30].

It has previously been reported that TAS2R-G_βγ_ signaling pathway-mediated VDLCC inactivation plays a pivotal role in chloroquine-induced relaxation [16]. We also found that inactivation of VDLCCs play a role in chloroquine-resulted relaxation (Figure 1).

In summary, chloroquine can induce relaxation in precontracted mouse airway smooth muscle through inactivation of VDLCCs and NSCCs. The inactivation of NSCCs is a result of direct blockade on the channels. Therefore, both VDLCCs and NSCCs should be blocked when treating airway hyperresponsiveness.
